# Infections and antimicrobial resistance in intensive care units in lower-middle income countries: a scoping review

**DOI:** 10.1186/s13756-020-00871-x

**Published:** 2021-01-29

**Authors:** Yulia Rosa Saharman, Anis Karuniawati, Juliëtte A. Severin, Henri A. Verbrugh

**Affiliations:** 1 Department of Clinical Microbiology, Faculty of Medicine, Universitas Indonesia/Dr. Cipto Mangunkusumo General Hospital, Jakarta, Indonesia; 2 Department of Medical Microbiology and Infectious Diseases, Erasmus MC University Medical Center Rotterdam, Dr. Molewaterplein 40, 3015 GD Rotterdam, The Netherlands

**Keywords:** Intensive care units, Bacterial drug resistance, Cross infection, Acinetobacter, Infection control

## Abstract

**Background:**

Intensive care units (ICUs) in lower-middle income countries (LMICs) are suspected to constitute a special risk for patients of acquiring infection due to multiple antibiotic resistant organisms. The aim of this systematic scoping review was to present the data published on ICU-acquired infections and on antimicrobial resistance observed in ICUs in LMICs over a 13-year period. A systematic scoping review was conducted according to the PRISMA extension guideline for scoping reviews and registered in the Open Science Framework.

**Main body of the abstract:**

Articles were sought that reported on ICU-acquired infection in LMICs between 2005 and 2018. Two reviewers parallelly reviewed 1961 titles and abstracts retrieved from five data banks, found 274 eligible and finally included 51. Most LMICs had not produced reports in Q1 or Q2 journals in this period, constituting a large gap in knowledge. However, from the reported evidence it is clear that the rate of ICU-acquired infections was comparable, albeit approximately 10% higher, in LMICs compared to high income countries. In contrast, ICU mortality was much higher in LMICs (33.6%) than in high income countries (< 20%). Multidrug-resistant Gram-negative species, especially *Acinetobacter baumannii* and *Pseudomonas aeruginosa*, and *Klebsiella pneumoniae* played a much more dominant role in LMIC ICUs than in those in high income countries. However, interventions to improve this situation have been shown to be feasible and effective, even cost-effective.

**Conclusions:**

Compared to high income countries the burden of ICU-acquired infection is higher in LMICs, as is the level of antimicrobial resistance; the pathogen distribution is also different. However, there is evidence that interventions are feasible and may be quite effective in these settings.

*Protocol Registration* The protocol was registered with Open Science Framework (https://osf.io/c8vjk)

## Introduction

**Fig. 1:**
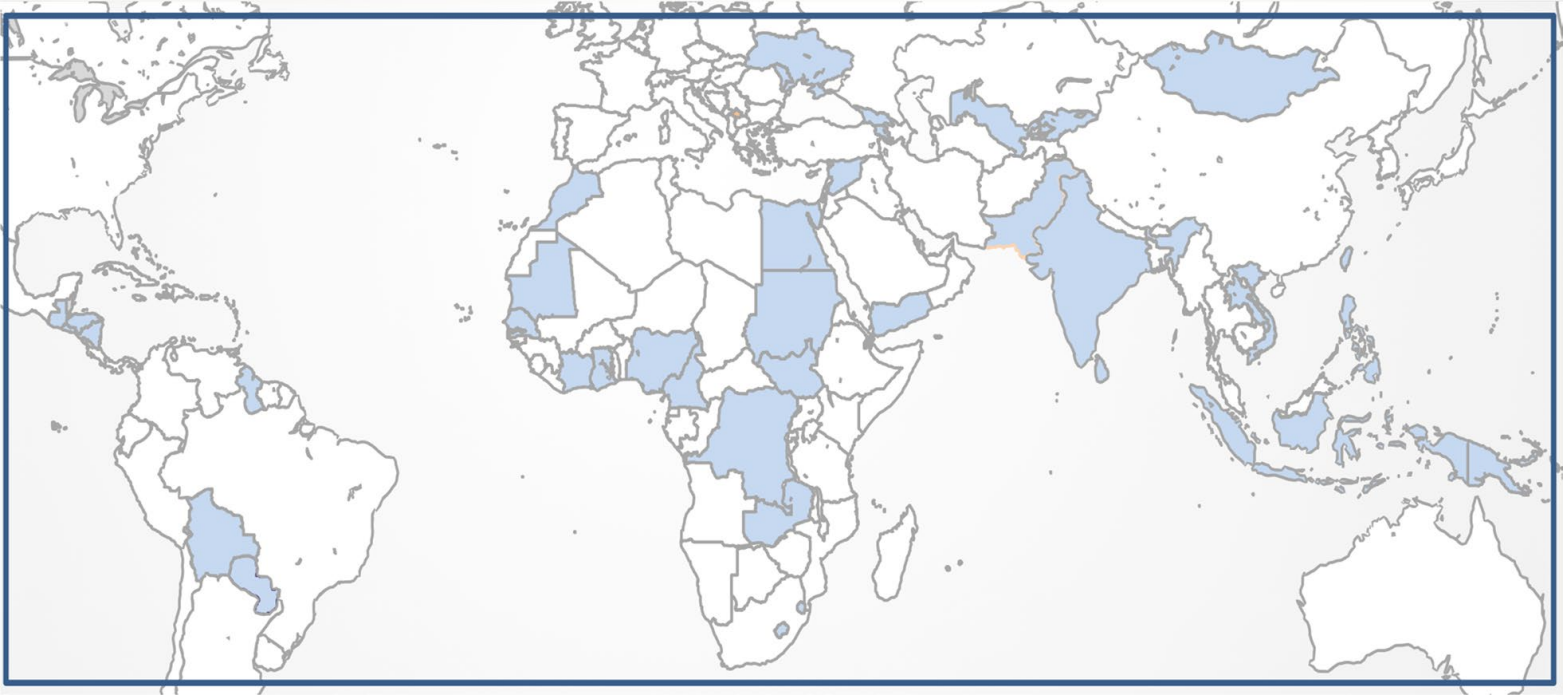
Global Map highlighting lower-middle income countries (blue)

Approximately fifty countries of the world belong to the category of lower-middle income countries (LMICs) according to the long-standing classification by the World Bank and updated every year [1]. These LMICs share the same bracket of Gross National Income (GNI) per capita—$1026 and $3955 (2019)—a proxy for the level of their economic progress. This LMIC group is a quite diverse group by region, size, population, and income level, ranging from tiny nations with small populations to giants like India and Indonesia (Fig. 1).

LMICs are known to be already affected by the worldwide pandemic of antimicrobial resistance. In the future, LMICs are considered to be at high risk of additional morbidity and mortality due to pathogens resistant to multiple antimicrobial agents as was stated in the report from the Wellcome Trust in 2014 [2]. Patients admitted to intensive care units (ICUs) are particularly at risk of acquiring infection due to multiple antibiotic resistant strains of notorious nosocomial pathogens including *Enterococcus spp., Staphylococcus aureus, Klebsiella pneumoniae, Acinetobacter baumannii, Pseudomonas aeruginosa* and *Escherichia coli* (a.k.a. the ESKAPE group of pathogens) [3]. A large international point prevalence survey on infections in the ICU conducted on May 8^th^, 2007 included 1265 ICUs in 75 countries and provided insight in the prevalence and outcomes of such infections [4]. However, only eight LMICs participated in that survey and data on the occurrence and determinants of ICU-acquired infections and antimicrobial resistant pathogens from LMICs remain relatively rare and published wide apart. We, therefore, present here a scoping review of the data published on the infections and antimicrobial resistance observed in ICUs in LMICs over a 13-year period and published in esteemed scientific journals. We focused on revealing which LMICs have produced relevant information in this period, and which not, what type of ICU infections were observed and at what frequencies, which species and types caused these ICU-acquired infections, and present their antibiotic resistance profile. In addition, information was sought about the role of healthcare workers (HCWs) and the ICU environment, and whether intervention studies were performed and, if so, successful in reducing (risk of) infections in these settings.

## Methods

### Protocol and registration

The scoping review protocol was developed as recently recommended by PRISMA extension for scoping reviews [5–7] and registered with Open Science Framework, an international prospective register of systematic scoping reviews on 13th December 2019 (https://osf.io/c8vjk) [5, 6].

### Eligibility criteria

Any study that targeted the etiology and management of nosocomial bacterial infections in adult ICUs in LMICs, with a focus on antimicrobial resistance and interventions applied were eligible. Also, results of screening for multidrug-resistant bacterial pathogens (ESKAPE species) among humans (patients and HCWs) and the hospital environment were considered eligible for inclusion in this review.

The population, intervention, comparison, and outcome (PICO) framework for determining the eligibility of the studies for the primary research question is presented in Table 1.

**Table 1:** Inclusion and exclusion criteria for this scoping review

Criteria	Inclusion	Exclusion
Population	Human	Animal, plants
	Adult	Children and neonates
	Intensive care units	Other hospital wards
	ICU infections, especially those acquired during ICU stay	
	Laboratory results of screening for the presence of multidrug-resistant bacteria, especially ESKAPE species among ICU patients, healthcare workers, or the ICU environment	
	Lower-middle income countries	
Intervention	Preventive measures to limit nosocomial acquisition and infection of bacterial pathogens	
Comparator	Not Applicable	
Outcomes	Infection and/or acquisition	
	Identification and susceptibility pattern of targeted pathogens (ESKAPE species)	
	Compliance with prevention protocols (e.g. hand hygiene)	
	Mortality	
	Length of stay	
Language	English Language	
Study design	Case control study	Editorials
	Cohort studies	Case series reports
	Cross-sectional studies	Conference abstracts/reports
	Longitudinal studies	Reviews
	Modelling studies	
	Laboratory-based studies	
Quality of journal	Q1 or Q2 based on rank on Web of Science	Q3 or Q4, or not ranked in Web of Science

### Information sources and search

We conducted a systematic scoping review of the epidemiology and management of multidrug-resistant bacteria in adult ICUs in countries classified as lower-middle income countries (LMICs) according to the World Bank (WB) in 2015. As stated by the WB, for the 2015 fiscal year, lower-middle income economies were those with a GNI per capita between $1026 and $4035. Thus, low income countries were not included in this review. The term country refers to any territory for which authorities report separate social or economic statistics.

The scoping review protocol was developed and registered in the Open Science Framework, an international prospective register of systematic scoping reviews, as recently recommended by PRISMA extension for scoping reviews [5, 6]. A systematic scoping review is a type of evidence synthesis method aimed at mapping the range of literature that exists around a specific topic of interest and focuses the research questions by charting the existing research findings and identifying research gaps. Scoping methodology is also considered a useful approach for determining the need and value of a future primary (in-depth study) or a full systematic review [5].

The review is restricted to papers published from January 1st 2005 until January 1st 2018, a time frame that is wide enough to allow all LMICs to contribute relevant data, and recent enough to still be relevant for today. We used EMBASE as the starting point and subsequently serially queried OvidSP ‘Medline’, Cochrane, Web of Science and finally Google Scholar. The relevant literatures were identified using a single-line search strategy [8] with the following search strings:

### Embase.com

('intensive care unit'/exp OR ((('intensive care' OR 'critical care') NEXT/1 unit*) OR icu OR icus):ab,ti) AND (infection/exp OR 'antibiotic resistance'/exp OR 'infection prevention'/exp OR 'infection control'/exp OR 'vancomycin resistant Enterococcus'/de OR 'methicillin resistant Staphylococcus aureus'/de OR 'extended spectrum beta lactamase'/de OR 'carbapenemase'/de OR 'Pseudomonas aeruginosa'/exp OR 'Acinetobacter baumannii'/exp OR (infection* OR sepsis OR septic OR nosocomial* OR mrsa OR ((multidrug OR multi-drug OR resistan*) NEAR/3 (bacter*)) OR ((vancomycin OR methicillin OR carbapenem) NEAR/3 resistan*) OR vre OR mrsa OR esbl OR (antibiotic* NEAR/3 resistan*) OR 'extended spectrum beta lactamase' OR 'extended spectrum β lactamase' OR 'Pseudomonas aeruginosa' OR 'Acinetobacter baumannii'):ab,ti) AND ((('lower middle' OR 'low middle' OR 'low- and middle') NEAR/6 income NEAR/3 countr*) OR lmic OR lmics OR Armenia* OR Mongolia* OR Bhutan* OR Morocc* OR Bolivia* OR Nicaragua* OR (Cabo NEXT/1 Verde*) OR Nigeria* OR Cameroon* OR Pakistan* OR Congo* OR ('Papua New' NEXT/1 Guinea*) OR 'Cote d Ivoire' OR Paraguay* OR Djibout* OR Philippin* OR Egypt* OR Samoa* OR Salvador* OR 'Sao Tome and Principe' OR Georgia* OR Senegal* OR Ghan* OR 'Solomon Islands' OR Guatemal* OR Guyana* OR (Sri NEXT/1 Lank*) OR Hondur* OR Sudan* OR India OR Swaziland* OR Indonesia* OR Syria* OR Kiribati* OR 'Timor-Leste' OR Kosov* OR Ukrain* OR Kyrgyz* OR Uzbek* OR Lao OR laos OR Vanuatu* OR Lesotho* OR Vietnam* OR Mauritania* OR ('West Bank' NEXT/2 Gaza) OR Micronesia* OR Yemen* OR Moldova* OR Zambia*):de,ab,ti NOT (((child/exp OR pediatrics/exp) NOT adult/exp) OR (pediatric* OR picu OR nicu OR picus OR nicus):ab,ti).

### Medline (OvidSP)

(Intensive Care Units/ OR (((intensive care OR critical care) ADJ unit*) OR icu OR icus).ab,ti.) AND (exp infection/ OR exp Drug Resistance, Microbial/ OR Vancomycin-Resistant Enterococci/ OR Methicillin-Resistant Staphylococcus aureus/ OR Pseudomonas aeruginosa/ OR Acinetobacter baumannii/ OR (infection* OR sepsis OR septic OR nosocomial* OR mrsa OR ((multidrug OR multi-drug OR resistan*) ADJ3 (bacter*)) OR ((vancomycin OR methicillin OR carbapenem) ADJ3 resistan*) OR vre OR mrsa OR esbl OR (antibiotic* ADJ3 resistan*) OR extended spectrum beta lactamase OR Pseudomonas aeruginosa OR Acinetobacter baumannii).ab,ti.) AND (((lower middle OR low middle OR low- and middle) ADJ6 income ADJ3 countr*) OR lmic OR lmics OR Armenia* OR Mongolia* OR Bhutan* OR Morocc* OR Bolivia* OR Nicaragua* OR (Cabo ADJ Verde*) OR Nigeria* OR Cameroon* OR Pakistan* OR Congo* OR (Papua New ADJ Guinea*) OR Cote d Ivoire OR Paraguay* OR Djibout* OR Philippin* OR Egypt* OR Samoa* OR Salvador* OR Sao Tome and Principe OR Georgia* OR Senegal* OR Ghan* OR Solomon Islands OR Guatemal* OR Guyana* OR (Sri ADJ Lank*) OR Hondur* OR Sudan* OR India OR Swaziland* OR Indonesia* OR Syria* OR Kiribati* OR Timor-Leste OR Kosov* OR Ukrain* OR Kyrgyz* OR Uzbek* OR Lao OR laos OR Vanuatu* OR Lesotho* OR Vietnam* OR Mauritania* OR (West Bank ADJ2 Gaza) OR Micronesia* OR Yemen* OR Moldova* OR Zambia*).kw,ab,ti. NOT (((exp child/ OR exp pediatrics/) NOT exp adult/) OR (pediatric* OR picu OR nicu OR picus OR nicus).ab,ti.)

### Cochrane

(((('intensive care' OR 'critical care') NEXT/1 unit*) OR icu OR icus):ab,ti) AND ((infection* OR sepsis OR septic OR nosocomial* OR mrsa OR ((multidrug OR multi-drug OR resistan*) NEAR/3 (bacter*)) OR ((vancomycin OR methicillin OR carbapenem) NEAR/3 resistan*) OR vre OR mrsa OR esbl OR (antibiotic* NEAR/3 resistan*) OR 'extended spectrum beta lactamase' OR 'extended spectrum β lactamase' OR 'Pseudomonas aeruginosa' OR 'Acinetobacter baumannii'):ab,ti) AND ((('lower middle' OR 'low middle' OR 'low- and middle') NEAR/6 income NEAR/3 countr*) OR lmic OR lmics OR Armenia* OR Mongolia* OR Bhutan* OR Morocc* OR Bolivia* OR Nicaragua* OR (Cabo NEXT/1 Verde*) OR Nigeria* OR Cameroon* OR Pakistan* OR Congo* OR ('Papua New' NEXT/1 Guinea*) OR 'Cote d Ivoire' OR Paraguay* OR Djibout* OR Philippin* OR Egypt* OR Samoa* OR Salvador* OR 'Sao Tome and Principe' OR Georgia* OR Senegal* OR Ghan* OR 'Solomon Islands' OR Guatemal* OR Guyana* OR (Sri NEXT/1 Lank*) OR Hondur* OR Sudan* OR India OR Swaziland* OR Indonesia* OR Syria* OR Kiribati* OR 'Timor-Leste' OR Kosov* OR Ukrain* OR Kyrgyz* OR Uzbek* OR Lao OR laos OR Vanuatu* OR Lesotho* OR Vietnam* OR Mauritania* OR ('West Bank' NEXT/2 Gaza) OR Micronesia* OR Yemen* OR Moldova* OR Zambia*):ab,ti NOT ((pediatric* OR picu OR nicu OR picus OR nicus):ab,ti).

### Web-of-science

TS = ((((("intensive care" OR "critical care") NEAR/1 unit*) OR icu OR icus)) AND ((infection* OR sepsis OR septic OR nosocomial* OR mrsa OR ((multidrug OR multi-drug OR resistan*) NEAR/3 (bacter*)) OR ((vancomycin OR methicillin OR carbapenem) NEAR/3 resistan*) OR vre OR mrsa OR esbl OR (antibiotic* NEAR/3 resistan*) OR "extended spectrum beta lactamase" OR "extended spectrum β lactamase" OR "Pseudomonas aeruginosa" OR "Acinetobacter baumannii")) AND ((("lower middle" OR "low middle" OR "low- and middle") NEAR/6 income NEAR/3 countr*) OR lmic OR lmics OR Armenia* OR Mongolia* OR Bhutan* OR Morocc* OR Bolivia* OR Nicaragua* OR (Cabo NEAR/1 Verde*) OR Nigeria* OR Cameroon* OR Pakistan* OR Congo* OR ("Papua New" NEAR/1 Guinea*) OR "Cote d Ivoire" OR Paraguay* OR Djibout* OR Philippin* OR Egypt* OR Samoa* OR Salvador* OR "Sao Tome and Principe" OR Georgia* OR Senegal* OR Ghan* OR "Solomon Islands" OR Guatemal* OR Guyana* OR (Sri NEAR/1 Lank*) OR Hondur* OR Sudan* OR India OR Swaziland* OR Indonesia* OR Syria* OR Kiribati* OR "Timor-Leste" OR Kosov* OR Ukrain* OR Kyrgyz* OR Uzbek* OR Lao OR laos OR Vanuatu* OR Lesotho* OR Vietnam* OR Mauritania* OR ("West Bank" NEAR/2 Gaza) OR Micronesia* OR Yemen* OR Moldova* OR Zambia*) NOT ((pediatric* OR picu OR nicu OR picus OR nicus))).

### Google Scholar

"intensive|critical care"|icu|icus infection|infections|nosocomial|mrsa|vre|esbl|"lower middle-income country|countries" |lmic|lmics|chine|egypt|indonesia|morocco|phillippines|algeria|bolivia|colombia|ecuador|guatemala|honduras|jamaica|nicaragua|thailand.

The references resulting from the Google Scholar data bank search were subsequently sorted by relevance, and only the first 200 references downloaded for inclusion [8].

### Study eligibility

**Fig. 2:**
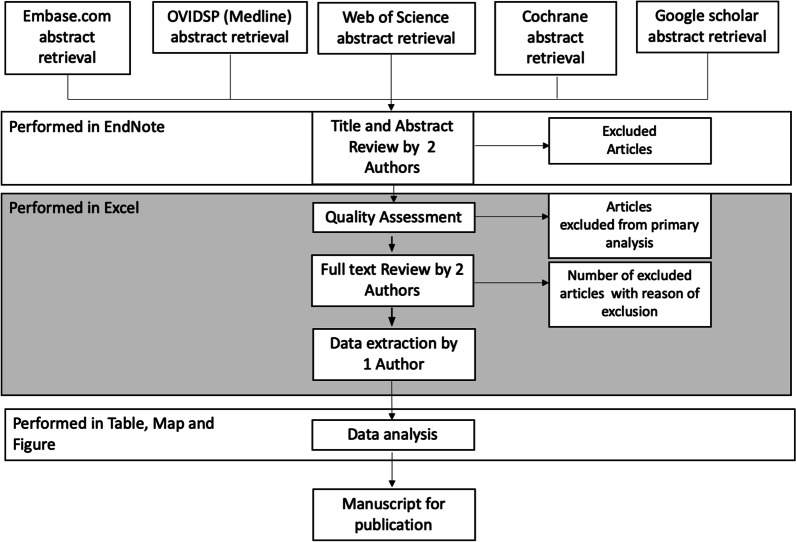
Overview of study methodology

We followed the outlined stages of study selection guided by the aforementioned eligibility criteria (Fig. 2). After retrieving by an experienced librarian, eligible papers (titles and abstracts) were exported to EndNote Library. The first author (YRS) screened all titles and abstracts and selected papers based on inclusion criteria. Another reviewer (HAV) independently performed a parallel review of titles and abstracts, and discrepancies between the two reviewers were resolved through consensus.

Subsequently, eligible papers published in journals ranked by their impact score as Q1 or Q2 in the Web of Science were selected for inclusion in the primary analysis. Full texts of the papers so selected were retrieved for full text review in a third round of screening for inclusion based on the criteria stated above, with reason for exclusion noted for each paper excluded on the basis of this full text review.

Papers excluded from the primary analysis based on the ranking of their journal of publication and those excluded during full text analysis were saved in separate files for potential analysis of specific questions arising during the remainder of the review process. Custom groups in EndNote were used to distinguish between various reasons for exclusion (Table 1), and articles were assigned to specific groups for certain sub-questions. The reviewers (YRS and HAV) worked in their own copies of this library. After reading all articles, each reference in the library was discussed in detail; therefore, no automatic comparison was used and any discrepancy was resolved [7].

### Data extraction

Data were extracted by first author (YRS) and inputted into a data extraction table (Excel) and independently checked by the senior author (HAV) to ensure quality.

The extracted data comprised the characteristics of each study (first author name, year of publication, country, study period and design), characteristics of hospital and adult ICUs, population characteristics, the type and characteristics of adult ICU-associated infection, laboratory diagnosis, the total and individual number of the species (Gram-negative and Gram-positive) isolated from patients, HCW screening and environmental screening, their phenotypic and genotypic resistance characteristic, and the outcomes of patients (see Table 1).

### Collecting and summarizing the findings

Thematic analysis was performed to identify the current etiology and management of nosocomial bacterial infections in adult ICUs in LMICs from the included studies. Where possible the results from the LMICs were compared with similar data collected from West-European countries in the same time frame. [4, 9–11].

## Results

### Study selection

**Fig. 3:**
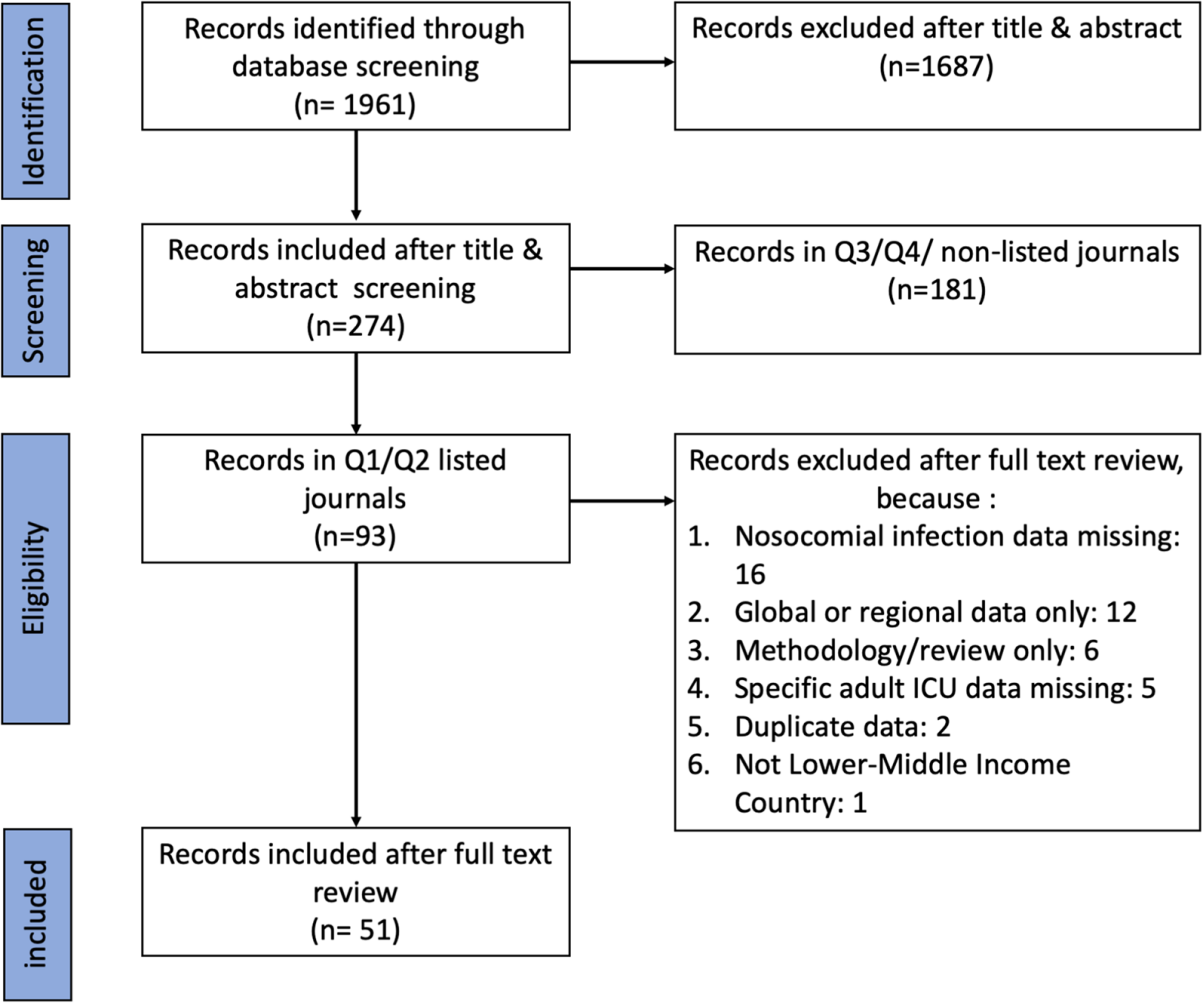
Flowchart for literature search

After duplicates were removed, a total of 1961 citations were identified from searches of electronic databases (Fig. 3). Based on the title and the abstract, 1687 were excluded, with 274 eligible articles published in journals ranked by their impact score by the Web of Science. Of these 274 articles, 93 were published in Q1 or Q2 journals and these 93 articles were subjected to a third round of eligibility check. Forty-two were excluded for specified reasons (see Fig. 3 for reasons of exclusion) and the remaining 51 papers were included in the primary analysis of this scoping review.

### Geographical distribution and characteristics of included studies

**Fig. 4:**
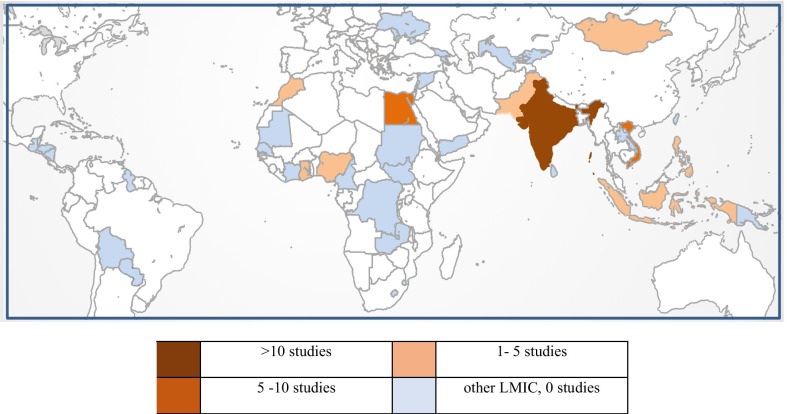
LMICs highlighted by number of studies reporting on Intensive Care Unit-associated infections in 2005–2018

All included studies were carried out in LMICs and were published between 2005 and 2018. Fifty-one qualified studies were conducted in South Asia (India: 22 studies [12–33], Pakistan: 2 [34, 35]), Middle East & North Africa (Egypt: 9 [36–44], Morocco: 2 [45, 46]), East Asia & Pacific (Vietnam: 6 [47–52], Indonesia: 2 [53, 54], Philippines: 2 [55, 56], Mongolia: 1 [57]), Sub-Saharan Africa (Nigeria: 2 [58, 59], Ghana: 1 [60]), and Europe & Central Asia (Kosovo: 2 [61, 62]) (Fig. 4). Thus, the large majority of LMIC did not have information on ICU-associated infections published in Q1 or Q2 journals in this time frame. Most publications described surveillance and observational studies, only ten publications reported on intervention studies, either randomized or quasi-experimental in design. Multicenter studies were described in 28 publications.

The characteristics of ICUs were not uniform, because some ICUs were highly specialized wards, including Burn ICU’s or Liver and post-transplant ICUs. However, most were mixed medical-surgical units with an open ward design. The number of beds per ICU ranged between 4 and 75 with a median (interquartile range [IQR]) size of 10 (8–15). The majority of patients were male (38–79%). Eleven studies presented the median of age of patients admitted, it ranged from 25 to 61 years, with a mean of the medians of 53 years. Twenty-three studies presented the mean age of patients admitted, it ranged from 32 to 71 years, with a mean of the means of 50 years. In the contemporary EPIC II study, the mean age of patients admitted to ICUs was 60.7 years [9].

### ICU infection rate and outcomes

The overall frequency of ICU infections was presented using three types of calculations, as an attack rate in 11 reports, as point prevalence in eight and as incidence rate in seven, with some reports using multiple measures (Table 2). The overall attack rate was 9.1 infections/100 admissions, and varied between 4.4 and 129.3/100 admissions [13, 15, 28, 36, 45, 49, 50, 58, 59, 61, 62]. We identified point prevalence data in 8 studies, overall it was 22.4 infected patients/100 admitted patients, and varied between 8.5 and 50 [13, 28, 42, 43, 53, 58, 59, 62]. The overall incidence rate was 9.1 infections/1000 patients days, based on data from 7 studies, it varied between 2.4 and 79 infections/1000 patients days in the ICU [13, 15, 28, 41, 43, 44, 59]. Expressed as device specific incidences ventilator-associated pneumonia (VAP) occurred at a rate of 11.3 episodes/1000 days on ventilation, central line-associated blood stream infection (CLABSI) at 4.1 episodes/1000 days with central line and catheter-associated urinary tract infection (CAUTI) at a rate of 3.0 episodes/1000 days with urinary catheter (Table 2).

**Table 2:** Infection rates in intensive care units in lower-middle income countries, 2005–2018

Biostatistical measure	Patients admitted to ICU	Patients infected during ICU stay	ICU-acquired infections	Total days stayed in ICU	Observed frequency	References
*Number*						
Attack rate	22,403		2032		9.1/100 admissions	[13, 15, 28, 36, 45, 49, 50, 58, 59, 61, 62]
Point prevalence	2129	476			22.4/100 admitted	[13, 28, 42–44, 53, 58, 62]
Incidence rate			3614	397,307	9.1/1000 ICU days	[13, 15, 28, 41, 43, 44, 59]
VAP rate			1404 VAP	124,393 days on ventilator	11.3/1000 days on ventilator	[14–16, 22, 25, 26, 28, 32, 38, 39, 41, 44, 48, 57]
CLABSI rate			1053 CLABSI	255,828 days with central line	4.1/1000 days with central line	[14–16, 21, 25, 28, 32, 33, 38, 39, 41, 44, 55, 57]
CAUTI rate			916 CAUTI	300,679 days with catheter	3.0/1000 days with catheter	[14–16, 25, 28, 32, 37–39, 41, 44, 48, 55–57]

The median lengths of stay were presented in 15 studies [13, 28, 30–32, 38, 47, 48, 50, 52, 54, 58–60, 62], it ranged between 5 and 17 days. We calculated an overall median of the medians length of stay of 11 days, and an overall mean of the medians length of stay of 10 days. The overall in-ICU mortality rate extracted from 18 studies was 33.6% (1753/5241) patients. If we looked at individual studies, we found a wide range in recorded mortality rates varying between 14 and 70% [13, 17, 20, 25, 30–33, 37, 41, 46, 52, 54, 59, 60].

### Etiology of infection acquired in ICU

Information on pathogens causing all ICU-associated infections was available from 11 studies [13, 16, 28, 32, 36, 43, 47, 50, 52, 58, 61], six studies included microbiological data specifically related to VAP [13, 14, 26, 28, 58, 59], seven had data related to CAUTI [13, 14, 28, 37, 56, 58, 59] and six had CLABSI data [13, 14, 21, 28, 58, 59].

**Fig. 5:**
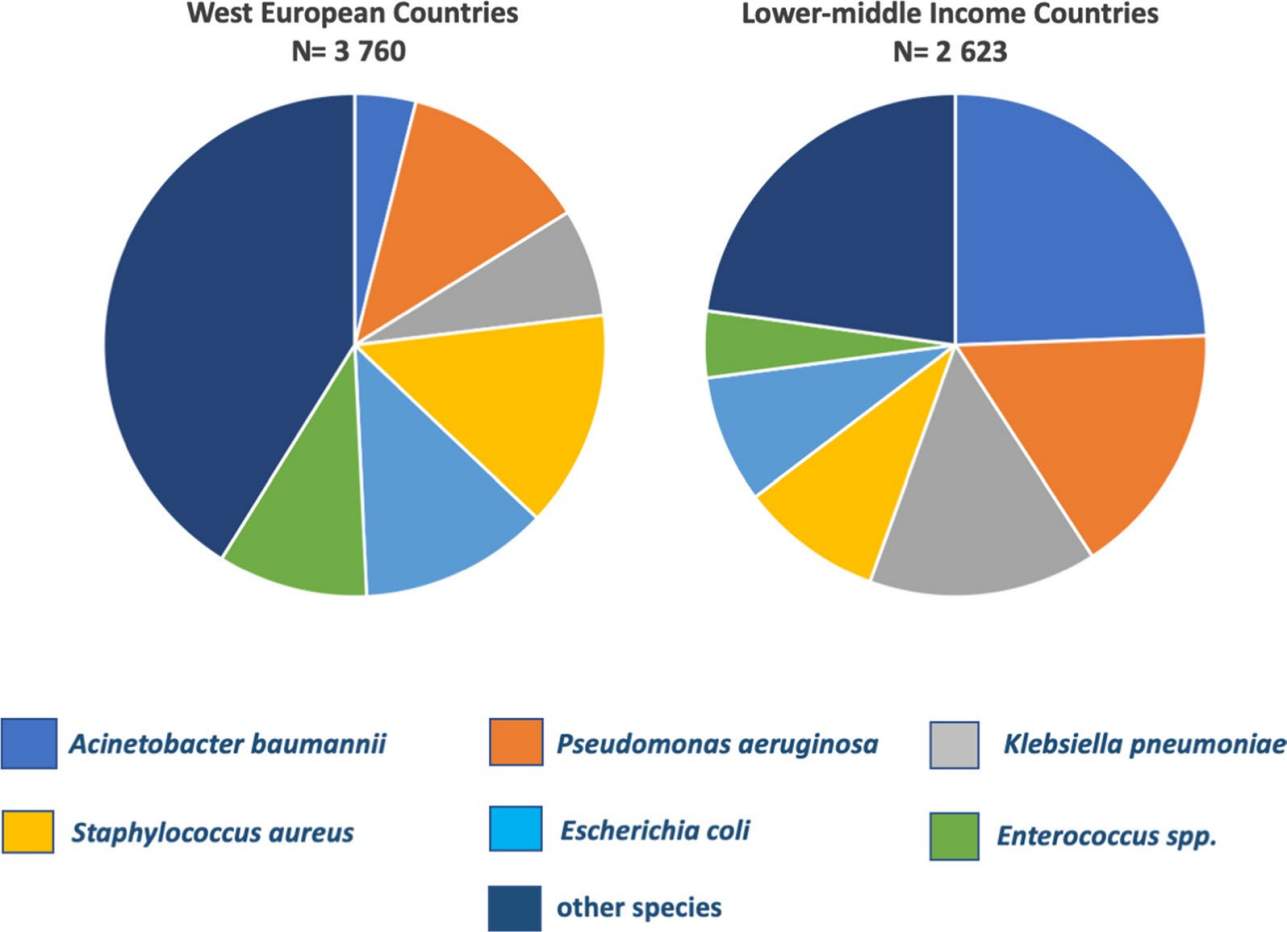
Distribution of ESKAPE pathogens causing ICU-acquired infection in LMICs and in West European countries. ESKAPE pathogens include *Enterococcus spp., Staphylococcus aureus, Klebsiella pneumoniae, Acinetobacter baumannii, Pseudomonas aeruginosa* and *Escherichia coli*. Data from West European countries were extracted from reference [9]

Gram-negative bacilli constituted the most prevalent group of nosocomial pathogens in these ICUs. The most common single pathogens causing ICU-acquired infection in LMICs were *A. baumannii* (24%)*, P. aeruginosa* (16%)*, K. pneumoniae* (15%)*,* these caused the majority of infections. This distribution of pathogens is significantly different from the distribution of pathogens causing ICU-acquired infection in West-European countries in the same period, where these same three species caused < 25% of all infections [9]. In the European setting, Gram-positive pathogens were more prominent and the group of other nosocomial agents of ICU infection was larger (Fig. 5).

**Fig. 6:**
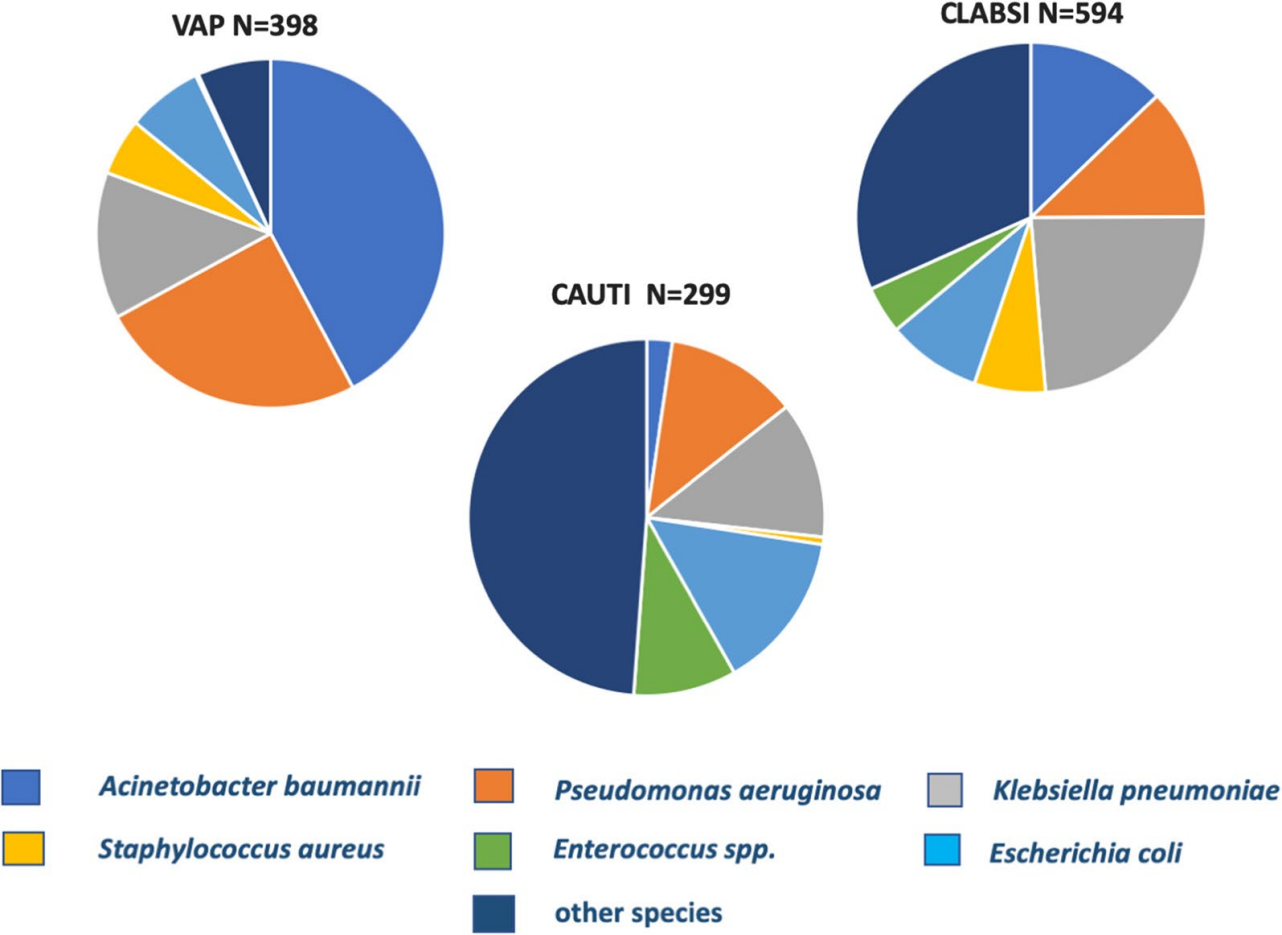
Distribution of ESKAPE pathogens causing Ventilator-Associated Pneumonia (VAP), Catheter-Associated Urinary Tract Infection (CAUTI) and Central Line-Associated Bloodstream Infection (CLABSI) in ICUs in lower-middle income countries, 2005–2018. ESKAPE pathogens include *Enterococcus spp., Staphylococcus aureus, Klebsiella pneumoniae, Acinetobacter baumannii, Pseudomonas aeruginosa* and *Escherichia coli*

*A. baumannii* was the most frequent pathogen identified for ventilator-associated pneumonia causing 42% of VAP, followed by *P. aeruginosa* which caused 25% of the VAP. Thus, these two species were involved in two thirds of all episodes of VAP in LMICs (Fig. 6). In contrast, *K. pneumoniae* was the dominant species in CLABSI, causing 24% of the episodes, as much as the combined impact of *A. baumannii* and *P. aeruginosa.* Together, the ESKAPE species were involved in two thirds of all CLABSI episodes. ESKAPE species also caused 51% of CAUTI in this setting, with *E. coli* as the most prevalent representative species. However, a sizable minority of CAUTI were caused by other species of uro-pathogenic microorganisms including many episodes that were caused by *Candida* species (data not shown).

### Phenotypic susceptibility pattern

Phenotypic resistance profiles of ESKAPE isolates to various antibiotics was determined in 15 studies. However, these studies were reported from only six LMICs, and were sometimes lacking data on certain combinations of ESKAPE species and classes of antimicrobial agents. Almost all isolates from LMICs were resistant to multiple classes of antibiotics, a condition that closely resembles the resistance patterns observed in most so-called Mediterranean countries, including Italy and Greece, located in the southern part of West-Europe (Table 3). Compared to isolates from LMICs, the same species isolated from invasive infections in Nordic countries of West-Europe, including Sweden and the Netherlands, displayed much lower levels of antibiotic resistances (Table 3). Vancomycin resistance among *Enterococcus* species was > 50% in Vietnam [50] and MRSA (methicillin-resistant *Staphylococcus aureus)* was identified in > 50% of all *S. aureus* isolates in most LMICs [28, 30, 35, 50, 59]. Multidrug resistant *K. pneumoniae, A. baumannii,* and *P. aeruginosa* were found among > 50% of the isolates in India, Pakistan, Egypt, Vietnam and Nigeria [13, 20, 27, 28, 35, 40, 42, 47, 52].

**Table 3:**
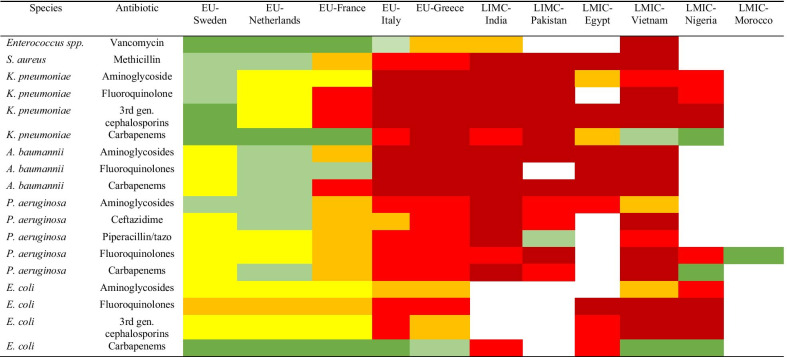
Phenotypic susceptibility patterns of ESKAPE species causing ICU infection in lower-middle income countries (LMIC) compared to susceptibilities of the same species causing invasive infections in indicated European Union (EU) countries, 2005–2018

Level of resistance: 
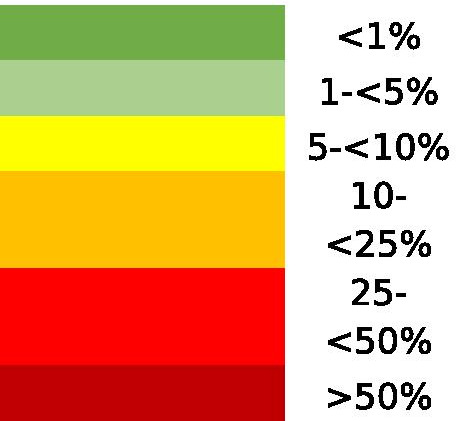
 Data from indicated European Union countries were derived from reference [11]. Colors indicate increasing levels of resistance as specified in the legend, and blank boxes indicate that no data was available for the particular combination of species and antimicrobial agent

### Genotypic resistance pattern

Only a very few studies presented genetic information regarding the antibiotic resistances observed. Amissah et al*.* from Ghana reported that 28% of isolates of *S. aureus* tested positive for the *mecA* gene [60]. Carbapenemase genes (*bla*_OXA-23,_ *bla*_OXA-51,_ *bla*_OXA-66,_ *bla*_OXA-68_) in *A. baumannii* were characterized in four studies, in Indonesia [54], Egypt [40, 42] and Morocco [46].

### Environment screening

Environmental screening cultures were performed and reported in four separate studies only. Taneja et al*.* in 2005 in India [12] collected 178 environmental samples from various sources and fluids in their main and transplant ICUs and found 51 (28.7%) to be contaminated with potential pathogens, of which 31 (17.4%) were contaminated with Gram-positive bacteria, 26 (14.6%) with Gram-negative bacilli and 11 (6.2%) with fungi.

Gupta et al*.* [27] more recently reported the presence of *A. baumannii* in 17/26 (65%) samples of humidifier water, and in 3/6 (50%) heat and moisture exchangers cultured in their ICU in a tertiary care center in South India. These environmental isolates showed the same multidrug resistance pattern as contemporary isolates from patients admitted to the ICU.

In Morocco, Uwingabiye et al. [46] identified 36 environmental *A. baumannii* isolates and compared them with 47 clinical isolates of the same species. They showed genetic similarity between the clinical and environmental isolates since 80/83 (96.4%) of all isolates belonged to the same 7 pulsed-field gel electrophoresis pulsotypes. Saharman et al. [54] likewise found six isolates of carbapenem-non-susceptible *A. baumannii-calcoaceticus* complex in the environment of two ICUs in a tertiary care center Indonesia, four of these isolates belonged to same dominant clone, defined by multilocus sequence typing, as those infecting their patients.

### Healthcare worker screening

HCWs may be another source of nosocomial pathogens, thus HCW screening may be an important measure to detect and eradicate such sources of antimicrobial resistance. However, only two studies addressing HCW carriage of resistant pathogens were available from LMICs in this time frame, one from Indonesia [54] and one from Ghana [60].

Saharman et al. [54] identified one HCW in their ICUs that carried a strain of carbapenem-non-susceptible *A. baumannii-calcoaceticus* complex*,* and Amissah et al*.* [60] found colonization with *S. aureus* isolates that were obtained from 13/29 (45%) of their HCWs, but only one of which carried MRSA.

### Intervention study

We identified 10 publications that described interventions aimed to reduce ICU-acquired infections and antimicrobial resistances; all but one applied a quasi-experimental design to measure the effects of their intervention (Table 4) [18, 21–24, 33, 34, 48, 49, 56]. Multimodal strategies (those with ≥ 3 components implemented in an integrated manner to achieve improved outcomes and change behavior as defined by WHO guidelines) were used in most studies [63]. Outcomes were either processes, especially hand hygiene (HH) practice, in five studies or they were actual rates of ICU-acquired infections in seven studies (two studies had both types of outcomes, Table 4). Thu et al*.* (2015) in Vietnam performed a cost-effectiveness study analyzing the impact of a HH improvement program in ICUs. The study used the steps recommended by the WHO, including upgrading HH facilities, training, surveillance, and feedback. The study showed that HH compliance increased from 25.7 to 57.5% and the incidence of HAI decreased from 31.7 to 20.3% (*p* < 0.001) after the intervention; similar results were shown in several reports from India [18, 23, 24].

**Table 4:** Intervention studies performed in lower-middle income countries, 2005–2018

Study	Study period	Published year	Country	Hospitals	ICUs	Objective	Study design	Intervention	Subjects or observations	Outcomes	Comments
Khan [34]	7/2006–11/2007	2009	Pakistan	1	1	Reduce VAP	Quasi-experimental before/after study	6 h training only	582 MV patients	VAP rate/100 MV patients from 18 to 13% (*p* = 0.11)	All patients were surgical and ventilated; MDR *A. baumannii, P. aeruginosa* and *K. pneumoniae* most prevalent
Mathur [18]	7/2010–9/2010	2011	India	1	1	Increase HH compliance	Quasi-experimental before/after study	Questionnaires, education & training, monitoring	1489 HH opportunities	Compliance from 8.4 to 63.1% (*p* < 0.0001). Housekeeping staff did not increase their HH compliance	Small scale, short term study; not clear whether housekeeping was trained or not
Jaggi [21]	9/2004–2/2012	2013	India	11	16	Prevent CLABSI by multidimensional approach	Quasi-experimental before/after study	Infection prevention bundle, education, monitoring & feedback	35,650 patients yielding 90,370 CL days	CLABSI/1000 CL days from 6.4 to 3.9 for a RR of 0.61 (0.46–0.81) *p* = 0.0007. Less *S. aureus* after intervention but more *P. aeruginosa. K. pneumoniae* most prevalent pathogen throughout study	Mean age was 1.3 year higher in intervention period
Mehta [22]	7/2004–10/2011	2013	India	14	21	Prevent VAP by multidimensional approach	Quasi-experimental before/after study	Infection prevention bundle, education, monitoring & feedback	46,945 patients yielding 65,574 MV days	VAP/100 MV days from 17.4 to 10.8 for a RR of 0.62 (0.50–0.78), *p* = 0.0001	Patients had little lower ASIS scores in intervention period
Navao-Ng [56]	12/2005–12/2010	2013	Philippines	2	4	Preventing CAUTI by multidimensional approach	Quasi-experimental before/after study	Infection prevention bundle, education, monitoring & feedback	3183 patients yielding 8720 UC days; observed HH opportunities 4191	CAUTIs/1000 UC days from 11.0 to 2.66 for a RR 0.24 (0.22–0.53); HH compliance from 57.2 to 78.2% (RR 1.37[1.21–1.54])	Few HH opportunities in baseline period
Schultsz [48]	5/2004–4/2006	2013	Vietnam	1	1	Prevent exogenous acquisition of MDRO	Quasi-experimental before/after study	HH reinforcement, revising infection prevention procedures, monitoring & feedback, adjust antibiotic policy	357 patients	VAP/1000 MV days from 56 to 40, UTI/1000 UC days from 12.8 to 15.0 (both not significant). Less cephalosporins, penicillin and carbapenem and more fluoroquinolones, metronidazole and broad-spectrum penicillin used; only MRSA acquisition delayed, not seen for other MDRO	Patients had more severe tetanus + more MV days + longer LOS in year 2; HH compliance only measured in year 2
Biswal [23]	11/2010–5/2013	2014	India	1	7	Improving HH	Quasi-experimental before/after study	Repeated education & training, posters, adequate supplies of alcohol & soap	3212 HH opportunities	HH compliance up from 16.5 to 28.2% and 35.1% after 1st and 2nd training week respectively. Significant in all ICUs	Low numbers of opportunities per ICU
Chakravarthy [24]	8/2004–7/2011	2015	India	3	3	Improving HH by multidimensional approach	Quasi-experimental before/after study	Allocation supplies, education & training, reminders, monitoring & feedback	3612 HH opportunities	HH compliance up from 36.9 to 74.8% for a RR 2.0 (1.7–2.4), *p* = 0.0001; but not in surgical ICU? poor among ancillary staff; HH improvement maintained over 3 years	Only few observations in surgical ICU
Thu [49]	6/2009–4/2011	2015	Vietnam	1	17	Reducing HAI by HH promotion	Quasi-experimental before/after	Questionnaires, education & training (including patients & visitors), posters & flyers, new sinks, hand alcohol made available	984 patients and 6046 HH observations	HAI/100 pts: from 31.7 to 20.3% (*p* = 0.005), all HAI types; HH compliance from 25.7 to 57.5% (*p* < 0.001)	
Rosenthal [33]	4/2012–8/2014	2015	India	2	5	CLABSI reduction	RCT, block-randomization	Introduced new IV flush device	1096 patients yielding 7680 CL days	CLABSI/1000d: 2.21 vs 6.40; RR 0.35 (0.16–0.76); cost effective, Qualys-increasing; shift in microbe species	

VAP (ventilator-associated pneumonia), MV (mechanical ventilation), RR (risk ratio), MDR (multidrug-resistant), CAUTI (catheter-associated urinary tract infection), UC (urinary catheter), HH (hand hygiene), MDRO (multidrug-resistant organism), CLABSI (central line-associated blood stream infection), CL (central line), HAI (hospital-acquired infection), RCT (randomized controlled trial), IV (intravenous)

Successful interventions have also targeted CLABSI, VAP, and CAUTI. The implementation of a multidisciplinary approach for prevention of VAP in ICUs in Pakistan [34] yielded a reduction from 18 to 13% in the VAP rate, and in India [22] VAP incidence decreased from 17.4 to 10.8 per 1000 ventilation days. In 16 ICUs in India a similar intervention strategy for CLABSI also showed a reduction in CLABSI incidence rates from 6.4 to 3.9 per 1000 central line days [21].

Finally, Navoa-Ng et al*.* in the Philippines targeted CAUTI and reported a reduction of CAUTI from 11.0 to 2.66 per 1000 urinary catheter days as a consequence of applying an infection prevention bundle together with education, monitoring and feedback [56].

### Additional information

Sixty-three papers were published in journals listed as Q3 or Q4 by the Web of Sciences but only nine [64–72] of those met our inclusion criteria after full text review. Those nine papers described six independent studies, all emanating from the countries already included in our primary analysis. The data extracted from those publications did not add novel information nor significantly changed the findings from our review of the information presented in our primary analysis. Specifically, the infection rates in these nine studies all fell within the range found in our primary analysis. In addition, only one paper from India [64] presented resistance rates of ESKAPE organisms; these rates all fell within the categories specified for India in Table 3. However, this paper also had resistance rates for *E. coli* against aminoglycosides, fluoroquinolones, and 3^rd^ generation cephalosporins, all were > 50%. Of note, Ikeh et al*.* reported [73] MRSA contamination of instruments and surfaces in an Nigerian ICU, and Joseph et al*.* [69] found evidence that some strains of *P. aeruginosa* and *A. baumannii* were shared between the ICU environment and patients, and one of 16 HCWs carried a *P. aeruginosa* strain that was shared with a patient that had developed VAP.

## Discussion

In this systematic scoping review, we have shown that endemic nosocomial infections represent a major burden and safety issue for patients admitted to intensive care in lower-middle income countries. Unfortunately, there were relatively few studies on this topic published from LMICs in a highly ranked scientific journals (Q1/Q2 by Web of Science). From 50 LMICs, we only identified 51 qualified published studies performed in 11 LMICs over a thirteen-year time frame. Supplementing information from studies published in Q3/Q4 journals was not helpful since only nine additional papers published in these journals met our inclusion criteria, and they did not expand the areas already covered. There is, thus, a great unmet need in most LMICs to perform surveillance of ICU-acquired infections and to characterize the nosocomial pathogens involved. Such data are needed in order to obtain a more comprehensive view and monitor the problems of LMICs to control ICU-acquired infection and combat resistance to antimicrobial agents in their settings.

The ICU-acquired infection rates were quite high in LMICs, with an average point prevalence rate of 22.4 infected patients per 100 present in the ICU. This rate is comparable, albeit somewhat higher, to the average point prevalence rate of 19.5% recorded in ICUs across West-European countries in the same time frame (2011–2012) [4]. The device-associated infection indices were also comparable to those recorded in West-European ICUs at that time, 9.5 VAP/1000 intubation days, 3.3 CLABSI/1000 days with central line and 4.5 CAUTI/1000 days with urinary catheter [10]. Thus, the overall impression is that ICU-acquired infections in LMICs are quite similar in their nature, but that rates are somewhat higher (approximately 15%) in LMIC ICUs compared to ICUs in West-European countries.

ICU length of stay and ICU mortality are important outcomes of intensive care. In studies retrieved by our search, the overall length of stay was 10—11 days and the overall ICU mortality rate was 33.6% (varying from 14 to 70% across the studies). In the same time frame in European countries, based on ICU surveillance from 2008 to 2012, the median (IQR) length of stay was 10 (8–12) days, which was highly comparable to the length of stay in LMICs [10]. However, mortality rates differed significantly. On average, 15.3% of EU patients staying more than two days died in the ICU, ranging from 8.7% in Luxembourg to 18.1% in France [10]. The Extended Prevalence of Infection in Intensive Care (EPIC II) study (2007) involving 1265 ICUs and 75 countries found an overall ICU mortality rate of 18.2% (2370/13,011 patients). Infected patients had higher ICU mortality rates (25.3%) and longer ICU lengths of stay (16 days [IQR, 7–34]) [9, 10]. Thus, the overall ICU mortality rate of 33.6% retrieved in this scoping review was much higher in LMICs, indicating that, compared to high income countries, patients in ICUs in LMICs die at a higher rate and that death comes relatively early during their ICU stay. The fact that in LMICs the mean age of ICU patients was much lower than in high income countries (50 years versus 60 years, respectively) further underscores the major discrepancy in ICU survival between these two groups of countries.

Gram-negative bacteria were responsible for more than 50% of the total number of ICU-acquired infections recorded in LMICs. This species distribution contrasts with findings from studies done in West-Europe at that time where the prevalent cause of healthcare-associated infections had switched over to Gram-positive microorganisms (72.7%) (EPIC II study) [9]. The microorganisms most frequently isolated from ICU infections in a later study [4] were in decreasing order, *E. coli* (15.9%), *S. aureus* (12.3%), *Enterococcus spp*. (9.6%), *P. aeruginosa* (8.9%), *Klebsiella spp*. (8.7%), coagulase-negative staphylococci (7.5%), *Candida spp*. (6.1%), *Clostridium difficile* (5.4%), *Enterobacter spp.* (4.2%), *Proteus spp.* (3.8%) and *Acinetobacter spp.* (3.6%) [4]. Especially the proportion of infections caused by *Acinetobacter spp.* in ICUs in LMICs was more than six times higher compared to West-European countries (24% versus 3.6%) [9].

The ESKAPE group of pathogens will be of increasing relevance to antimicrobial chemotherapy in the coming years. Our findings revealed a high rate of multidrug-resistant (MDR) Gram-negative bacilli causing ICU infections in LMICs. The high proportions of strains resistant to third generation cephalosporins and of multidrug resistance among Gram-negative bacteria are especially worrisome. Comparably high rates of MDR among Gram-negative bacilli isolated from patients with invasive infections have been reported from Italy, Greece and some in France (EARS-Net by 30 EU/EEA countries in 2014) [11]. In contrast, much lower MDR rates among Gram-negative bacilli were observed from invasive infections in Sweden and the Netherlands [11]. The high percentages of resistance to carbapenems of *P. aeruginosa, A. baumannii* and *K. pneumoniae* isolates found in this scoping review reflect the challenges of treatment of ICU patients in LMICs. Although not reported in the studies included in this review the determinants of antimicrobial resistance in LMICs are likely to include a high selection pressure due to overconsumption of antibiotics and the lack of barriers against the spread of selected resistant clones in healthcare settings.

The implementation of a multidisciplinary approach for prevention of HAIs in ICUs from LMICs showed that reductions in the HAI rate are possible in LMICs. Some studies reported effective interventions including contact precautions, active surveillance cultures, monitoring, audit and feedback of preventive measures, patient isolation or cohorting, HH improvement programs, and environmental cleaning. This is also highlighted by the recent evidence-based WHO Guidelines on the core components of IPC programs, which strongly recommend multimodal strategies to translate IPC measures into clinical practice [63].

One of the most comprehensive guidelines is the 2013 European Society of Clinical Microbiology and Infectious Diseases (ESCMID) Guidelines for the management of infection control measures to reduce transmission of multidrug-resistant (MDR) Gram-negative bacteria [74]. In endemic settings, HH and contact precautions were the only two interventions that were strongly recommended for all three pathogens (MDR-*K. pneumoniae*, MDR-*P. aeruginosa*, and MDR-*A. baumannii*) in addition to isolation for MDR-*K. pneumoniae* and isolation, alert codes, education, and environmental cleaning for MDR-*A. baumannii*. In epidemic settings, hand hygiene, contact precautions, active screening, isolation and, last but not least, environmental cleaning are strongly recommended for all three pathogens in addition to alert codes and cohorting for MDR-*K. pneumoniae *[74]. Interestingly, implementation of HH best practices and environmental cleaning was reported in only few studies in LMICs so far. Effective HH compliance is widely recognized and strongly recommended by WHO to reduce transmission of pathogenic microorganisms in healthcare. Likewise, the important role of the innate environment of the ICU providing sources and routes of transmission of MDR microorganisms is gaining recognition worldwide. This scoping review revealed that implementation of these guidelines is essentially possible in LMICs, and are sorely needed to reduce the high burden of disease caused by ICU-acquired infections in these settings. Much room for further high quality observational and interventional research remains that should include more countries with a LMIC status, and target novel interventions that are cost-effective in this particular setting.

The ICU cannot be rendered sterile but every effort should be made to reduce the number of ICU-acquired healthcare-associated infections (HAI) and the risk of spread of resistant nosocomial pathogens. Strategies to minimize infection have been incorporated into various guidelines on ICU design that are available in the UK, the USA and Europe [75, 76]. An ICU should accommodate at least 6 beds with 8–12 beds considered as the optimum. Hospitals with several smaller units should be encouraged to rearrange these units into a single larger department to improve efficiency. A larger ICU may provide opportunities to create separate, specialized functional subunits with 6–8 beds, sharing the same geographical, administrative, and other facilities [75, 76]. However, of those included in this review most ICUs in LMICs still had open ward designs with one large room, with beds separated by curtains only, if at all, they did not have separate cubicles or separate isolation rooms. The numbers of beds ranged between 4 and 75 beds. These open ICU designs are not optimal, they compare unfavorably with the current trend to construct ICUs as a series of separate rooms to better protect patients against ICU-acquired infections [75]. Thus, the roles of the environment and of HCWs in the endemicity and transmission of nosocomial pathogens in ICU settings should be further studied and delineated, they should no longer be underestimated. Also, not all ICUs in LMICs had dedicated and qualified intensivists; however, most of them did have multidisciplinary teams in charge of the patients (data not presented).

A limitation of this review is posed by the relatively low number of qualified studies that were performed in only a minority of the 50 countries belonging to the group of LMICs. We also restricted our review to publications in the English language. Although the vast majority of medical and healthcare research is published in English, we may have missed important information from researchers that elected to publish their data in another language. Thus, this review cannot be taken to reflect the full scope of ICU-acquired infections in all LMICs, but from our perspective this currently represents the best available view on infections acquired in ICUs and the species and resistance profiles of the organisms causing such infections in LMICs.

## Conclusions

Our systematic scoping review describes the current evidence of ICU-acquired infections in LMICs. Many gaps in knowledge remain since most LMICs have not produced high quality reports. However, from the reported evidence it is clear that the rate of ICU-acquired infections is likely to be somewhat higher in LMICs compared to high income countries and that the ICU mortality rate is much higher. MDR Gram-negative bacilli, especially *Acinetobacter spp.* and *Pseudomonas spp.* from the environment clearly play a much more dominant role in LMICs than in high income countries. However, interventions to improve this situation have been shown to be feasible and effective, even cost-effective.

## Data Availability

The datasets used and/or analysed during the current study are available from the corresponding author on reasonable request.
